# Porous safety net: catastrophic health expenditure and its determinants among insured households in Togo

**DOI:** 10.1186/s12913-018-2974-4

**Published:** 2018-03-12

**Authors:** Esso-Hanam Atake, Djesika D. Amendah

**Affiliations:** 1 University of Lomé (Togo), Lomé, Togo; 2 African Population and Health Research Center, APHRC Campus, Kirawa Road, P.O. Box 10787 – 00100, Nairobi, Kenya

**Keywords:** Catastrophic health expenditure, Compulsory health insurance scheme, Insured households, Illness, Hospitalization, Togo

## Abstract

**Background:**

In Togo, about half of health care costs are paid at the point of service, which reduces access to health care and exposes households to catastrophic health expenditure (CHE). To address this situation, the Togolese government introduced a National Health Insurance Scheme (NHIS) in 2011. This insurance currently covers only employees and retirees of the State as well as their dependents, although plans for extension exist. This study is the first attempt to examine the extent to which Togo’s NHIS protects its members financially against the consequences of ill-health.

**Methods:**

Data was obtained from a cross-sectional representative households’ survey involving 1180 insured households that had reported illness in the household in the 4 weeks preceding the survey or hospitalization in the 12 months preceding the survey. The incidence and intensity of CHE were measured by the catastrophic health payment method. A logistic regression was used to analyse determinants of CHE.

**Results:**

The results indicate that the proportion of insured households with CHE varies widely between 3.94% and 75.60%, depending on the method and the threshold used. At the 40% threshold, health care cost represents 60.95% of insured households’ total monthly non-food expenditure. This study showed that the socioeconomic status, the type of health facility used, hospitalization and household size were the highest predictors of CHE. Whatever the chosen threshold, care in referral and district hospitals significantly increases the likelihood of CHE. In addition, the proportion of households facing CHE is higher in the lowest income groups. The behaviour of health care providers, poor quality of care and long waiting time were the main factors leading to CHE.

**Conclusion:**

A sizable proportion of insured households face CHE, suggesting gaps in the coverage. To limit the impoverishment of insured households with low income, policies for free or heavily subsidized hospital services should be considered. The results call for an equitable health insurance scheme, which is affordable for all insured households.

## Background

About 150 million households worldwide face catastrophic health expenditure (CHE) annually, and some 100 million were driven into poverty because of health care costs, according to the World Health Organization (WHO) estimates about a decade ago [1]. These numbers were bound to increase over the last decade in the absence of major changes in the health financing system. Indeed, the fifty-eighth World Health Assembly made a call to “plan the transition to universal coverage of population” which theoretically should decrease exposure to CHE. This call resonates in Sub-Saharan Africa (SSA), where households’ protection from the consequences of health shocks is a major concern because of the importance of household out-of-pocket payments (OOP) at the point of service as a means of financing the health system [2].

Recently, considerable literature has emerged around the theme of health insurance, universal coverage and CHE. Wagstaff and Doorslaer [3] have shown that the incidence and intensity of “catastrophic” payments—both in terms of pre-payment income as well as ability to pay—were reduced between 1993 and 1998, and that both incidence and intensity of “catastrophe” became less concentrated among the poor in Vietnam as a result of change in implementation of insurance policy. Recently, Kusi et al. [4] found that the Ghanaian national health insurance scheme has significantly reduced out-of-pocket (OOP) payments and offered financial protection against CHE for insured individuals and their households. Also, it was found that Rwanda Mutual Health Insurance Scheme led to a fourfold decrease in incidence of CHE [5]. Other studies have also shown that a well-functioning prepayment system has a strong potential to improve financial protection against illness [6–8]. In contrast, a few studies have suggested that insurance is not a panacea against CHE. According to Ekman [9], health insurance did not provide financial protection against the risk of catastrophic payments in Zambia using 1998 data; on the contrary, insurance was found to increase such risk. China’s New Cooperative Medical System program between 2004 and 2007 clearly did not meet one of its key goals of providing insurance against catastrophic illnesses [10].

In West Africa, the introduction of insurance and fees waiving schemes has not shielded all households from substantial out of pocket expenditure. In Nigeria, despite the implementation of the National Health Insurance Scheme (NHIS) to protect families from the financial hardship, a study based on Oyo State has shown low uptake and significant CHE among the poorest quintile [11]. In Mali, the State implemented a fee waiver for Caesarean sections and a maternity referral-system; however those policies fail to eliminate financial obstacles for remote households so that women who underwent Caesareans continue to incur catastrophic expenses [12]. Also, studies in Burkina Faso showed that rich enrolees in the Community-Based Health Insurance Programs used more health care and faced less CHE than poor enrolees [13–15].

In Togo, a West African country, neighbour to Burkina Faso, Ghana and Benin, household contributions to total health expenditures accounted for 48.6% in 2012 [16]. This proportion is more than twice as high as the 15%–20% threshold recommended by the WHO [17, 18]. To address the substantial out-of-pocket payments and thus the CHE problem, the Togolese government introduced a National Health Insurance Scheme (NHIS) in 2011. The establishment of the NHIS in 2011 is a major step by the Togolese government to address the perverse problems of the “cash and carry” system. However, in practice, insured households experience difficulties accessing care with their NHIS because of the health professionals’ and facilities’ reluctance to deal with the insurance’s heavy administrative procedures and delayed reimbursement [19]. In addition, the NHIS covers only a predetermined list of medications or formulations at pre-set prices to contain cost; thus, reducing patients and their providers’ options. Oftentimes, listed drugs are stocked out, prices changed [19] without due notice. A satisfaction survey of NHIS enrolees conducted in 2015, revealed a list of issues: dissatisfaction with management of chronic diseases, coverage exclusion, long waiting times, informal payments to providers, and the heavy claim procedures [20].

In this context, it may be possible that insured households still face important OOP leading to CHE. This paper seeks to examine the incidence and intensity of catastrophic payments and determinants of CHE among households insured NHIS in Togo. Empirical evidence on CHE and its determinants among insured households may provide useful information to policy makers to improve services offered by Togo’s compulsory health insurance scheme. This paper also adds to the growing body of international research which seeks to examine the incidence and intensity of catastrophic payments and its determinants for the achievement of meaningful universal coverage [21–24]. This paper is part of a series of studies on influence of health insurance on households’ economic well-being.

### Overview of Togo’s National Health Insurance Scheme (NHIS)

Since 2010, Togo implemented a new approach to national planning for health. A new National Health Policy and a National Health Development Plan for 2012–2015 was drafted in 2011 [25]. Along the same lines, legislation calling for the establishment of a national health insurance scheme targeting civil servants, central administration staff, local collectivities, para-public agencies, and retired public sector workers was passed in 2011 [26]. Fees for caesarean sections were waived, the same year [26]. In 2012, the National Health Insurance Scheme (NHIS), began paying for the health services, covering approximately 300,000 people [25]. The scheme is managed by the National Institute of Health Insurance (INAM). NHIS aims to provide quality health care along with financial protection to enrolled households by covering risks associated with diseases, non-occupational accidents and maternity. NHIS is a mandatory health insurance which covers civil servants, civil servant retirees, and up to five dependents—spouse and four children aged 21 or under. The rate of coinsurance paid by members varies depending on the type of service [27]. Coverage of the private and informal sectors through NHIS has not begun yet. The care package covers different proportions of costs depending on the condition/disease. For instance, the NHIS covers 80% of the cost of general and specialized consultations, pharmaceutical medicines, laboratory tests and medical imaging, nursing care, orthopaedic-devices; 90% of surgeries and other inpatient care and 100% of pregnancy and childbirth care. Family planning commodities and services are not covered. This mandatory health insurance scheme is financed by monthly premium contributions set at 7% of the basic monthly salary split equally between the state employer and the main insured person. Services in public health facilities are covered by INAM; private health facilities, pharmacies, and eye care facilities can apply for accreditation [27]. It is estimated that in 2016, 196 private facilities were accredited [25].

## Methods

### Measuring catastrophic health expenditure (CHE)

Various methods have been used to evaluate the importance of health expenditure with respect to the total household income, household expenditure, household consumption, and household expenditure net of spending on basic necessities. Each method has its advantages and drawbacks [3, 28–30].

Two approaches of CHE were used in this study: capacity to pay (CTP), and total expenditure. CTP is defined as the share of household expenditure net of spending on basic necessities [3, 28–31]. In our study, we used food expenditure at subsistence level as a proxy for basic necessities [28, 31]. Two indicators were used: the catastrophic payment headcount (incidence) and the catastrophic payment gap (intensity). The catastrophic payment gap captures the average severity of payment above the catastrophic threshold [31].

### Statistical analyses

#### Variables

The variables (listed below) and computational steps to generate them are summarised here:

- *Q* = Total expenditure of household
- *T* = Household out of pocket payment for health care
- *D*(*Q*) = Total food expenditure of household
- *Y* = Household’s capacity to pay
- *z* = A specific threshold (from 5% to 40%). It represents the point at which the absorption of households’ resources by health care is considered to impose a severe disruption to their living standards [28].
- *H* = Incidence of CHE (catastrophic payment headcount)
- *OOP*ratio = Ratio of out of pocket health expenditure to total household’s expenditure
- *E* = Indicator variable which equals 1 if a household (i) is classified as having incurred CHE
- *G* = Catastrophic payment overshoot
- *MPO* = Mean positive overshoot
- *N* = Sample size

### Steps

#### Step 1

Compute household’s capacity to pay by subtracting total food expenditure of household from total expenditure of household:

$$ Y=Q-D(Q) $$

#### Step 2

OOP health payments share of household capacity to pay is defined as the ratio of OOP payments to the household’s capacity to pay:

$$ OOP\mathrm{ratio}=\raisebox{1ex}{$T$}\!\left/ \!\raisebox{-1ex}{$Y$}\right. $$

#### Step 3

The CHE is computed as a binary = 1 if household incurred catastrophic expenditure, and 0 otherwise. For a given threshold *z*(from 5% to 40%), the CHE would be obtained as follow:

$$ {E}_i=\left[\begin{array}{l}1\  if\  OO\mathrm{Pratio}-z\ge 0\\ {}0\  if\  OO\mathrm{Pratio}-z\prec 0\end{array}\right] $$

#### Step 4

The incidence of CHE (catastrophic payment headcount) is measured as the proportion of the sampled households whose OOP health expenditure as a fraction of their total non-food expenditures exceeds the specified threshold (*z*). The catastrophic payment headcount is estimated as: 92560995

$$ H=\frac{1}{N}\sum \limits_{i=1}^N{E}_i $$

#### Step 5

The catastrophic payment overshoot which measured the intensity of the catastrophic OOP health expenditure is estimated as:

$$ G=\frac{1}{N}\sum \limits_{i=1}^N{O}_i $$ with *O*_*i*_ = *E*_*i*_(*T*_*i*_[*Q* − *D*(*Q*)] ≻ *z*).

#### Step 6

The MPO which measures the mean overshoot among households making CHE at a specified threshold in the sample is estimated by related the incidence (*H*)and the intensity or overshoot (*G*). The MPO is calculated as follow:

$$ MPO=\frac{G}{H} $$

To check the robustness of the results obtained using the above methodology, we use the total expenditure approach. All indicators above can also be defined with total expenditure of household (*Q*)as denominator.

### Measuring determinants of CHE

The logistic regressions model [32] was used to analyse the determinants of CHE. The dependent variable takes the value of 1 if health care costs as a share of CTP exceed the threshold level (i.e. if the household experienced catastrophic health expenditure) and 0 otherwise.

### Explanatory variables chosen

With regards to the explanatory variables, the literature suggests that the likelihood of a household incurring CHE is a function of its socio - demographic characteristics such as the size, age of head of household, household head’s educational level, place of residence, health status of household members, economic status of the household, and type of health facility used [4, 7, 8, 33–35]. Kusi et al. [4] showed that, in Ghana, households which experienced hospitalisation of a member were over 7.0 times more likely than those without hospitalisation to incur CHE. In Iran, 42.6% of hospitalized subjects encountered catastrophic health expenditure [36]. Studies showed that hospitalization, household members with chronic illness and poverty status of the household are the major factor determining the financial catastrophe related to ill health [4, 36, 37]. The likelihood of facing CHE were 4.4. and 27 times higher among households having chronically illness persons and those that had case of hospitalization, in Georgia [37]. Along the same lines, Buigut et al. [6] support that health expenditure depends on the type of illness and where health service is sought.

In this study, owing to multicollinearity, the explanatory variables used were: type of illness, health status of household members, hospitalization, type of health facility used, household size, age of head of household, and income quintile. We expected the hospitalization and health status of household members (chronic illness) to be positively correlated with catastrophic expenditure. We assumed that households facing catastrophic expenditure are affected by type/place of treatment they receive [4, 34, 37]. Moreover, we hypothesized that household size and age of head of household affect positively CHE. Studies have shown that, in Burkina Faso, only household size, among households characteristics had a positive association with CHE at 30% and 40% threshold level [34]. It has been demonstrated also that catastrophic expenditure were more common among the households who had more elderly people [38] as individuals spend more on healthcare with increasing age [39, 40]. All these variables are described in Table 1.

**Table 1:** description of explanatory variables

Variables	Description
Illness and treatment	
Type of illness	Variable with several modalities 1. Respiratory/pneumonia illnesses; 2. Malaria/fever; 3. Skin disease; 4. TB; 5. HIV/AIDS; 6. Diabetes; 7. Diarrhoea; 8. Intestinal worms; 9. Infections and injuries, 10. Cancer; 11. Hypertension; 12. Sickle Cell; 99. Others
Having a family member with a chronic illness	Binary variable = 1 if a household member has any of the following diseases: Hypertension, Diabetes, Cardiac disorders, Arthritis, HIV /AIDS, ulcers, Gout, Cancer, asthma, sinusitis, and Sickle Cell; 0 otherwise
A family member was hospitalized the last 12 months preceding survey	Binary variable = 1 if a family member was hospitalized the last 12 months preceding survey; 0 otherwise
Health System/community	
Type of health facility used	Variable with several modalities 1.Self-medication, 2. Traditional healers 3. Reference hospitals 4. Dispensaries 5. District hospitals 6. Private health facilities
Distance to seek health care The health facility used is it close to place of residence?	Binary variable = 1 if the health facility used is close to place of residence; 0 otherwise.
Characteristics of the household	
Household size	Continuous quantitative variable
Age of head of household	Variable with 3 modalities 1. (18–49); 2. (50–60); 3. (60 and older)
Economic status of household	
Income group	Income quintile 1. Lower 2 3 4 5. Higher

### Sampling and data collection

This paper used the cross-sectional representative insured household survey data of monitoring of NHIS project implemented by the Centre de Recherche et de Formation en Economie et Gestion (CERFEG) in partnership with African Population and Health Research Center (APHRC).

### Study area

In Togo, a West African country, the incidence of poverty varies from 33% in Lomé-commune the capital city to 91% in the Northern Savanah region [41]. The country is divided into 6 administrative health regions, comprising forty (40) health districts and more than 882 peripheral health units. Lomé-Commune comprises about a quarter of the national population (24%), but three-quarter (76%) of private health facilities and physicians (74%) and the highest concentration of insured households (40%) [42].

### Sample size

To define the sample size for data collection, we used the Lwanga and Lemeshow approach [43]. With a 95% confidence interval for a difference in proportions, sample size is given by:

5 $$ \left({p}_1-{p}_2\right)\pm 1.96\sqrt{\frac{p_1\left(1-{p}_1\right)}{n_1}+\frac{p_2\left(1-{p}_2\right)}{n_2}} $$

Where *p*_1_ represents the incidence of CHE among publicly insured households, and*p*_2_the incidence of CHE among privately insured households. *n*_1_ and *n*_2_ are the groups sample size.

We relied on several recent studies carried out in developing countries to estimate the true proportion of *p*_1_ and *p*_2_. Barros et al. [44] found that among households with private insurance in Brazil, CHE varied from 2% to 16%. In twelve Latin American and Caribbean countries, the percent of households with CHE ranged from 1 to 25% [45]. In Ghana, Togo’s western neighbour which shares many of the same ethnic groups and potentially the same health behaviour, the incidence of CHE among fully insured households which sought healthcare from national health insurance system was 6.0% [4]. In contrast, Onwujekwe et al. [46] showed that about 27% of insured households incurred CHE, in four local government areas in southeast Nigeria. We therefore conservatively hypothesize that the CHE among the public and private insured households are respectively around 25% and 15% in Togo.

With absolute precision of 5%, the required sample size is 484 in each group, thus 968. We increased the sample size by 22% to account for sampling errors, mistakes in filling questionnaires and data entry mistakes, as well as fair management of interviewers (number of interviews per person). The final sample interviewed was 1180 insured households.

### Data collection

We designed a questionnaire based on the World Health Survey 2002 and the latest Togo’s 2013 Demographic Health Survey questionnaires. Trained interviewers conducted in-person face-to-face interviews. To minimise recollection errors, data were collected exclusively from insured households with at least one case of illness in the 4 weeks preceding the survey or at least one case of hospitalization the 12 months preceding the survey.

Lomé-Commune is organized in 5 health districts with different population size and density. To obtain a representative sample of Lome-Commune, we accounted for density by district as determined by the latest Togo Population and Housing Census [47]. The numbers of insured households surveyed in each district are respectively 36, 490, 264, 84, and 306. In each district, we randomly chose a street and sent interviewers along the road. They went into every household and interviewed only the consenting heads of insured households or their spouses in May 2016. This study focused only on 578 households insured with NHIS.

### Main variables construction

Data collected included health utilization and spending, household income and expenditure, education, occupation and marital status, gender, age, and other socioeconomic background information about insured households. We used the exchange rate of 16 May 2016 which is the middle of the month and is about the average of the period 1 USD = 579 XOF.

Appropriate reference periods were used to collect the expenditure data. All expenditures were adjusted in the same unit of 4 weeks. For example, during data collection, the reference period for clothing, shoes, maintenance and major repair expenses was the 3 months preceding the survey, while that of expenses such as electricity, water, etc. was 1 month preceding the survey [6]. This difference in reference period is explained by the fact that electricity and water bills tend to be incompressible and are paid monthly, while expenses for clothing and shoes are more discretionary and much less regular. Thus, we divided clothing expenditure by three. With regards to food expenditures, we first collected details on the previous day food consumption, then all information on food expenses for the previous 7 days preceding the survey [6]. We then extrapolated the weekly food consumption expenditures to 4 weeks. Food expenditure include cereals, vegetables, tubers, fruits, spices, fish, meat, oil and fat, milk and eggs, restaurant meals, etc. As for health expenditure, collected information includes expenses for medicines and vaccines, diagnostic and laboratory tests fees, consultation and treatment costs, hospitalization fees, cost of visits to traditional healers, transport, and other expenses related to outpatient care over the last 4 weeks preceding the survey; similar expenses were collected for hospitalization but with a year reference period.

## Results

### Descriptive statistics

#### Socio-demographic characteristics of the insured households

Table 2 summarizes socio-demographic characteristics of the sample. Most insured households were headed by men (72.39%) who were married (84.99%) and aged between 18 and 49 years (86.11%). About six out of ten household heads of households in our sample had some university level education (59.23%), while 29.89% had dropped out of high school. For a smaller percentage, the highest education levels were junior high school (7.93%) and primary education (2.21%). Most households had between 2 and 4 persons (45.05%), followed by households consisting of 5 or 6 persons (23.06%), households consisting of 1 or 2 persons (17.16%) and finally, households consisting of more than 6 persons (12.73%).

**Table 2:** Socio-demographic characteristics

Variables	Frequencies (%)
Characteristics of household head	
Age	
18–49	465 (86.11)
50 -	65 (12.04)
60 -	10 (1.85)
Gender	
Female	148 (27.61)
Male	388 (72.39)
Marital status	
Married/living together	453 (84.99)
Divorced/separated	19 (3.56)
Widower/widow	16 (3.00)
Unmarried	45 (8.44)
Educational level	
No formal schooling	2 (0.37)
Preschool	2 (0.37)
Primary	12 (2.21)
Junior secondary school	43 (7.93)
Senior secondary school	162 (29.89)
High level (university)	321 (59.23)
Household size	
1–2	93 (17.16)
3-	255 (45.05)
5 -	125 (23.06)
7 -	69 (12.73)
Income quintile	
1	106 (20.54)
2	118 (22.87)
3	94 (18.22)
4	96 (18.60)
5	102 (19.77)

The distribution of NHIS beneficiaries by wealth quintile shows that the households in the lower wealth quintiles represent around 33.37%; the tops quintiles are around 50% of insured households. The analysis of the income level by quintile reveals startling inequalities: insured households belonging to the fifth highest quintile have an average annual income per capita almost 4 times as high as that of the lowest quintile.

#### Diseases/conditions, provider of health care and distribution of expenses

Malaria was the main and the most frequently reported disease (46.18%), followed by infections and injuries (6.45%), intestinal worms (5.69%), and hypertension (4.93%). Other reported diseases included diarrhoea (3.04%), respiratory diseases/pneumonia (3.04%), and diabetes (2.28) (Table 3). These results seem to be consistent with Togo’ national health statistics. In Togo, the most frequently reported disease are malaria (44.9%), infections and injuries (8.4%), intestinal worms (4.9%), hypertension (1.8%) [48] in 2015.

**Table 3:** Use of health care and household’s expenditure

Variables	Frequencies (%)
Use of health care	
Types of illness/Symptom	
Malaria	230 (46.18)
Infection/injuries	34 (6.45)
Intestinal worms	30 (5.69)
Hypertension	26 (4.93)
Others	66 (9.17%)
Source of treatment	
Self-medication	161 (29.93)
Traditional healers	17 (3.16)
Referral hospitals	78 (14.50)
Dispensaries	67 (12.45)
District hospitals	129 (23.98)
Private health facilities	85 (15.80)
Household’s expenditure	
Mean OOP health expenditure (medical & nonmedical) (FCFA)*	29, 434.2
Mean household food expenditure (FCFA)	106, 675.3
Mean household non-food expenditure	204, 206.2
Mean household total expenditure	310, 881.5
Concentration Index (retirees and the still -employed)	0.0063

*The FCFA is the name of the currency used in part of West African countries including Togo

About 29.93% of insured households used self-medication in case of illness. About a quarter (23.98%) received care from district hospitals and 14.50% from referral hospitals. However, it should be emphasized that more than half (50.93%) of the insured households used public health facilities compared to 15.80% who used private ones. Finally, a small proportion (3.16%) had recourse to traditional healers.

The mean monthly expenditures of insured households were at 310, 881.5 FCFA (or 536.928 USD using the average value for the period reference). The mean OOP, including medical and non-medical expenditures, was 29, 434.2 FCFA (50.836 USD) or about 9.47% of the total expenditures. The average health expenditure per household represents 14.41% of total expenditure net of food spending.

#### Incidence and severity of CHE among insured households

Table 4 presents the incidence (headcount) and intensity (overshoot) of CHE. For sensitivity analysis, we varied the threshold from 5% to 40%. The 29% of the sample who used self-medication decided themselves to support OOP. Thus, they are excluded from the household facing CHE.

**Table 4:** Proportion of households experiencing catastrophic health expenditure

Threshold level, z (%)	5	10	15	25	30	40
Model 1 Capacity to Pay (CTP): Out-of-Pocket (OOP) as percent of total monthly non-food expenditure						
% Head count	75.60 (0.43)	54.60 (0,498)	36.48 (0.482)	21.01 (0.408)	14.70 (0.354)	9.71 (0.296)
% Overshoot	12.46 (0.1734)	9.18 (0.162)	6.92 (0.148)	4.09 (0.120)	3.24 (0.106)	2.03 (0.082)
% Mean Positive Overshoot	16.48 (0.182)	16.82 (0.189)	18.97 (0.194)	19.51 (0.197)	22.03 (0.191)	20.95 (0.177)
Model 2 OOP as percent of total monthly expenditure						
% Head count	64.30 (0.480)	33.07 (0.471)	19.95 (0.4)	11.02 (0.313)	8.13 (0.274)	3.94 (0.195)
% Overshoot	7.11 (0.136)	4.74 (0.124)	3.47 (0.111)	2.07 (0.087)	1.59 (0.078)	1.03 (0.061)
% Mean Positive Overshoot	11.06 (0.156)	14.33 (0.182)	17.41 (0.195)	18.75 (0.199)	19.53 (0.202)	26.08 (0.173)

(): standard deviation

In model 1, we used the capacity to pay (total monthly non-food expenditure) as a reference. The proportion of insured households facing CHE decreased from 75.60% to 9.71% as the threshold increases from 5% to 40%. The mean positive overshoots (MPOs) shows that the insured households facing CHE at the 30% threshold on the average spent 52.03% (30% + 22.03%) of their total monthly non-food expenditure on health care. For households incurring CHE at the 40% threshold, health care cost represents 60.95% (40% + 20.95%) of their total monthly non-food expenditure. These households incurring CHE at the 40% threshold represent about 9.71% of the sample.

Using monthly expenditure, we found a similar trend as observed in Model 2 but with smaller magnitudes. The proportion of households facing CHE decreased from 64.30% to 3.94%, as the threshold increased from 5% to 40%. At the 40% threshold, households with CHE spent 66.08% of their total monthly non-food expenditure on health care.

Comparing retirees and the still-employed by the government, the concentration index was 0.0063 indicating a very slight inequality between both groups.

#### Determinants of catastrophic health expenditure

In this section, we analyse factors associated with CHE among insured households with a logistic regression. First, we examined Variance Inflation Factors (VIF) for multicollinearity in our sample. The largest VIF was 8.74 and the mean VIF was around 1.98, which does not suggest high multicollinearity. We used the Hosmer-Lemeshow to test assess goodness of-fit [49] and found it satisfactory (Table 5). In conformity with WHO’s approach [1, 50], we estimated the model 1 with OOP as percent of total monthly non-food expenditure with thresholds of 10, 20, 30, and 40%.

**Table 5:** Determinants of CHE based on OOP as percent of total monthly non-food expenditure

Variables	Odds ratio (robust std. error)			
	10%	20%	30%	40%
Age of household head (15–49 years reference)				
50–59	0.52(0.53)	0.37(0.46)	−0.32(0.59)	−0.83(0.95)
60 – and above	−0.74(0.92)	−0.17(0.99)	− 0.24(1.02)	−0.15(1.30)
Household size	0.26(0.10)^a^	0.16(0.13)	0.32(0.22)	− 0.01(0.14)
Income group (1 = lowest reference)				
2.	0.26(0.45)	0.24(0.53)	1.27(0.77)^a^	0.17(0.88)
3.	0.52(0.44)	0.35(0.54)	1.32(0.72)^a^	0.77(0.81)
4.	−0.11(0.45)	0.48(0.52)	−0.23(0.73)	− 0.34(0.89)
5. higher	−0.31(0.53)	− 0.54(0.52)	− 0.35(0.65)	− 0.09(0.73)
A family member was hospitalized the last 12 months preceding survey				
1. Yes	0.84(0.35)^b^	1.53(0.38)^a^	2.10(0.44)^a^	2.96(0.62)^a^
Distance to health facility				
1.Yes	0.69(0.40)^c^	−0.24(0.37)	0.12(0.46)	0.66(0.65)
Having a family member with a chronic illness				
1. Yes	0.13(0.33)	0.71(0.32)^b^	0.46(0.38)	0.68(0.52)^a^
Type of health facility used (traditional healers = reference)				
Referral hospitals	2.14(0.39)^c^	1.44(0.43)^c^	1.21(0.50)^b^	1.34(0.71)^a^
Dispensaries	0.93(0.41)^b^	−0.56(0.73)	−0.73(1.02)	− 0.87(1.47)
District hospitals	1.96(0.33)^c^	1.43(0.42)^c^	1.00(0.54)^a^	1.88(0.65)^c^
Private health facility	1.92(0.35)^c^	1.04(044)^b^	0.22(0.64)	0.71(0.79)
Pseudo R2	0.1021	0.1457	0.2031	0.2853
H-L goodness-of-fit test	0.1970	0.0961	0.1176	0.2203

^a^significant at 1%; ^b^significant at 5%; ^c^significant at 10%; *H-L*: Hosmer-Lemeshow

The results indicate that hospitalization significantly impacts the likelihood of experiencing CHE across all thresholds. But other socio-demographic characteristics such as household size and income indicate mixed results. At 10%, any increase in household size by one leads to an increase in the likelihood of CHE by 14% but at 40%, the direction of the association is significantly opposite. Similarly, the impact of the household wealth quintile on CHE is mixed. We found statistically significant results at the 30% threshold where households in the two lowest quintiles face a higher likelihood of CHE. The signs of the coefficients show that the likelihood of CHE is higher among households in lowest incomes group, and that this likelihood decreases as the income increases.

In other words, the likelihood of CHE increases with hospitalization and decreases with income. Similarly, the likelihood of CHE increases significantly with access to hospital care. At the lower thresholds of 10 and 20%, the likelihood of CHE is higher in referral hospitals and district hospitals, compared to private health facilities. At 30% and 40% thresholds, only the use of referral hospitals and district hospitals positively affects CHE. Whatever the chosen threshold, care in referral and district hospitals significantly increases the likelihood of CHE. Furthermore, recourse to traditional healers significantly increases OOP with a higher likelihood compared to private health facilities.

## Discussion

This study is probably the first to attempt to examine the extent to which Togo’s national health insurance system protects its members financially against ill-health. The results suggest that a substantial proportion of insured households still face CHE, but the incidence of CHE is sensitive to the method and the threshold used.

At the 10% threshold, the proportion of insured households which faced CHE was 54.60%. At the 40% threshold (commonly used in the literature), this proportion fell to 9.71%. This incidence of CHE is well above similar studies carried out in Ghana (6.00%), India (4.8%), Vietnam (4.2%), and Nepal (4.56%) [4, 7, 51, 52]. These results are particularly concerning since at 30% and 40% thresholds, health care cost represents 55.03% and 60.95% of the households’ total monthly non-food expenditure respectively. These high proportions of households’ non-food expenditure allocated to health care could result from asset sales, reduction of savings, indebtedness and impoverishment of insured households in Togo [24, 53–55]. We hypothesize that the high proportion of CHE may also be due to several factors: some conditions or formulations of prescription are excluded; also, some insured households may be paying out-of-pocket rather than asserting their insurance right, as providers devote more time and energy to those uninsured patients who pay cash the costs of health care directly rather than those with insurance who required heavy administrative procedures before reimbursement.

Regarding determinants of CHE, this study showed that socioeconomic status, type of health service sought, hospitalization, and household size are the main determinants of CHE.

One interesting finding is that the importance of household income in predicting CHE. CHE decline as income rises, regardless of the threshold used. These results are consistent with those of Su et al. [34] who showed that in Nouna District in Burkina Faso (Togo’s northern neighbour), the proportion of households facing CHE is higher in the lowest income groups, whatever the threshold used. These results are also in agreement with those obtained by Buigut et al. [6] who showed that in Kenya, households in the highest income tertile were less likely to experience CHE compared to those in the lowest income tertile. Considering the higher proportion of poorer households’ income or consumption allocated to food, any small expenditure on health can be financially disastrous to them. It is thus difficult for the poorest of the poor to divert resources from basic needs [56]. We could conclude that high income level affords some protection against CHE [57–59]. These results suggest that, to limit the impoverishment of insured households with low incomes, policies of free or highly subsidized hospital care should be considered, at least for the poor. The results call for an equitable health insurance scheme and affordable for all insured households [6].

The importance of the household size is mixed. At the 10% threshold, the results suggest that the household size increases the likelihood of the CHE occurrence. Large households are significantly associated with high CHE in other studies as well [33, 34, 60]. However, at the 40% threshold, the results are inversed. In Kenya, Buigut et al. [6] found that increase in the household size decreases probability of CHE.

Another important finding was that hospitalization significantly increases the likelihood of CHE. The likelihood of CHE is low among insured households without hospitalization, irrespective of the chosen threshold. These results matched of those of Anbari et al. [36] who found that hospitalization was one of the highest predictors of facing CHE, in Iran. There are several possible explanations for these results. First, more severe illnesses require hospitalization and patients might require medication or formulation that are not covered by the insurance or that providers do not offer at the listed prices, requesting the difference from the patient. Moreover, hospitalization increases transport cost for the family members and thus direct non-medical spending. Third, the fee-for-service structure of the insurance may favour supplier-induced demand that raises expenses including those not covered by the insurance. Indeed, the relationship between care provider and patient is characterized by asymmetric information, the care provider may increase the demand for care and therefore the increase in the financial burden borne by the patient. These results corroborate those of Mbaye et al. [61] and Haddad et al. [62] who studied the policies of subsidized C-section childbirths in Senegal and in Benin respectively. In those two countries in order to protect families against CHE, costs associated with C-section deliveries are subsidized by the State, while those of normal delivery are not. The authors found an increase in the likelihood of C-sections in Senegal and an increase in the hospital length of stay in Benin. Those changes generate additional revenues for providers and contribute to higher costs for both insurer and insured. These results suggest a need for improvement of the NHIS’s implementation in terms of strengthening functions and the means of regulation and control.

It is interesting to note the significant association between the type of health care facility and CHE. The likelihood of CHE is higher with care in referral and district facilities which are publicly owned and are expected to be more affordable than the private health facilities. A possible explanation might be that patients in referral hospitals are sicker and receiving specialized and expensive care including: emergency or intensive care, surgery services etc. These referral hospitals care for most serious or severe cases across the country. We however hypothesize that one of the key explanations is the behaviour of health care providers towards insured households (care provider/patient relationship). It is generally found that care providers devote more time and energy to uninsured patients who pay the costs of health care directly rather than those with an insurance which requires cumbersome administrative procedures before reimbursement. Thus, in order to receive any immediate and comprehensive care, good quality health care and more attention, some insured households prefer OOP to asserting their insurance right. These results suggest, firstly, the need to sensitize health care providers on issues of equity in access to health care and the impact of health care cost on household impoverishment, particularly poor households. Additionally, the relationship between the NHIS, health facilities, and health care providers must be reviewed and improved, by taking into account the constraints and challenges facing health care providers.

It is interesting to note that longer distance to seek health care contribute to high CHE due to transport cost. This result may be explained by the fact that transport cost is not included in the benefit package of the NHIS. Any policies reducing the physical barrier to health care could help reduce the CHE [4].

Finally, the households’ health care seeking behaviour may also affect the cost of treating the disease, as fall back to traditional healers significantly increases the likelihood of CHE, particularly for low-income households. As in Ghana [4], a possible explanation might be that traditional healers’ care is not covered by NHIS. Given the importance of this sub-sector, the State should consider the possibility of a health insurance system extending its services to practitioners of alternative medicine recognized or approved by the State [4].

### Limitation

This study has some limitations. The expenditure data used to measure the different indicators were self-reported and not verifiable with other administrative sources. Moreover, the recall period of health expenditure data was 4 weeks, much longer than the two recommended 2 weeks for outpatient but less than 3 months or a year used for other studies [6, 63]. So, we cannot ignore the potential for measurement error in the assessment of OOP and CHE. Furthermore, because of the problem of multicollinearity, it was impossible to analyse the relationship between gender, educational level, marital status and CHE. We assumed that educational level and marital status could be approximated by the level of income and household size.

Despite these limitations, this study has several strengths. It was based on a random sampling of insured households which help with generalizability; it provided useful information on gaps in coverage among insured households and raised other questions. For instance, further studies should compare incidence and determinants of CHE among households with different types of insurance, NHIS and other private insurance.

## Conclusion

By examining the incidence and intensity of CHE and by identifying the main factors associated with CHE, this study aims at analysing financial protection against the disease risk of households insured by NHIS in Togo.

Our results indicate that households enrolled in the State mandatory scheme for its employees and their dependent NHIS are not fully protected against CHE. Factors such as hospitalization, type of health facility used, household income, and household size are significantly associated with CHE. Several policy implications emerge from our study.

(i) Medical care fee exemption or heavier subsidies for low-income insured households should be considered to ensure affordability and equity for all insured households
(ii) The relationship between the state compulsory insurance, care providers and patients should be re-examined in order to reduce asymmetric information and provide better quality health care at a lower price.

## Abbreviations

APHRC: African Population and Health Research Center
CERFEG: Centre de Recherche et de Formation en Economie et Gestion
CHE: Catastrophic health expenditure
CTP: Capacity to pay
MPO: Mean positive overshoot
NHIS: National health insurance scheme
OOP: Out-of-pocket payments
SSA: Sub-Saharan Africa
VIF: Variance inflation factors
WHO: World Health Organization
